# Effects of Different Pre-Heating Welding Methods on the Temperature Field, Residual Stress and Deformation of a Q345C Steel Butt-Welded Joint

**DOI:** 10.3390/ma16134782

**Published:** 2023-07-02

**Authors:** Jie Yuan, Hongchao Ji, Yingzhuo Zhong, Guofa Cui, Linglong Xu, Xiuli Wang

**Affiliations:** 1 College of Mechanical Engineering, North China University of Science and Technology, Tangshan 063210, China; yuanjie99530@163.com; 2 School of Materials Science and Engineering, Zhejiang University, Hangzhou 310030, China; wangxl@zju.edu.cn; 3 China MCC 22 Group Corporation Limited, Tangshan 063035, China; zhongyingzhuo@126.com (Y.Z.); 18000351356@163.com (L.X.); 4 Tangshan Longquan Machinery Co., Ltd., Tangshan 063300, China; 13832829121@163.com

**Keywords:** ceramic heating, flame heating, butt welding, residual stress, finite element simulation

## Abstract

Heavy plate welding has been widely used in the construction of large projects and structures, in which the residual stress and deformation caused by the welding process are the key problems to address to reduce the stability and safety of the whole structure. Strengthening before welding is an important method to reduce the temperature gradient, control the residual stress and reduce the deformation of welds. Based on the ABAQUS software, the thermal elastoplastic finite element method (FEM) was used to simulate the welding thermal cycle, residual stress and deformation of low-alloy, high-strength steel joints. Based on the finite element simulation, the influences of flame heating and ceramic heating on the temperature field, residual stress distribution and deformation of a Q345C steel butt-welded joint were studied. The results showed that the thermal cycle of the ceramic sheet before welding had little influence on the whole weldment, but had great influence on the residual stress of the weldment. The results show that the maximum temperature and residual stress of the welded parts are obviously weakened under the heating of ceramic pieces, and the residual stress of the selected feature points is reduced by 5.88%, and the maximum temperature of the thermal cycle curve is reduced by 22.67%. At the same time, it was concluded that the weld shapes of the two were basically the same, but the weld seams heated by ceramic pieces had a better weld quality and microstructures through comparing the macro- and micro-structures between the welded parts heated by ceramic pieces and the simulated weld. Heating before welding, therefore, is an effective method to obtain a high weld quality with less residual stress and deformation.

## 1. Introduction

Q345-series steel accounts for the largest proportion in the output of thick plate factories and covers the largest range of varieties and specifications. Under the new equipment conditions, it is a technical problem that most thick plate enterprises are concerned with maximizing the potential performance [1].In the construction and manufacturing of complex large-scale projects and structures, welding is the main assembly and connection process [2].The strength of a welded joint determines the service quality and service life of the whole structure. The magnitude and distribution of residual stress in the component or structure affects the subsequent machining and the life prediction and evaluation of structural reliability [3,4]. Excessive welding deformation will reduce the dimensional accuracy of welding and increase additional stress [5]. It is an urgent task, therefore, to find a reasonable and effective way to reduce the residual stress and deformation.

Due to the complexity of the welding process, more and more scholars have made use of the possibility provided by modern software to conduct numerical simulations of the welding and heat treatment processes. A numerical simulation is an effective tool to study the welding process and to evaluate the mechanical properties of welded parts [6]. The finite element analysis welding method is a powerful and reliable technique to observe the temperature field and stress field in the welding process and to predict the residual stress and deformation in a welding structure, which can directly show the performance of a welded joint [7,8]. Yegaie et al. [9] used the finite element method to build a three-dimensional heat engine model in order to predict the temperature and residual stress distribution in the process of gas-shielded tungsten welding (GTAW) with a radiator. According to the experimental data, it was verified that the high temperature area was only near the heat source, and low levels of longitudinal and transverse residual stress were generated near the welding area. In order to find an effective optimization method for welding deformation, Islam et al. [10] developed a numerical optimization framework based on a coupled genetic algorithm (GA) and finite element analysis (FEA), and they performed a classical weakly-coupled thermo-mechanical analysis using thermos-elastoplastic assumptions to predict the deformation of numerical models. The results showed that the proposed framework can significantly improve the quality of final welding products. Ai et al. [11] built a 3D numerical simulation model to study the weld characteristics generated in fiber laser keyhole welding. Based on the numerical simulation results, the evolution of the weld contour features was demonstrated, and the results were used to estimate the shape and size of the generated weld. Kik et al. [12] presented the calibration of the numerical model for the multi-layer welding of X22CrMoV12-1 steel. The calibration of the heat source model based on the actual geometric size and single pass shape allowed the accurate determination of process parameters, and they obtained results that were highly consistent with the actual welding tests. However, many research studies have only focused on the simulation analysis of the single pass welding of a thin plate, while research on the temperature field, stress field and post-welding deformation of a thick plate in the multi-layer and multi-pass welding processes are relatively few.

Among the many factors affecting welding behavior, the structure of the pre-welding member [13], parameters of welding [14], selection of a heat source [15], welding methods and optimization [16] and the treatment method after welding [17,18], affect the quality of welding, the service life of a component and the safe operation of it. These factors are the guarantee of a high-performance weld and good mechanical properties [11,19,20]. In addition to focusing on the change of the heat input conditions, the study also obtained the microstructure through a pre-welding heat treatment, thereby obtaining a moderate hardness and good weld to enhance the service life and reduce cracks [21]. Peng et al. [22] studied the effect of a pre-welding heat treatment on the microstructure and mechanical properties of an electron beam-welding IN738LC superalloy. The results revealed that the pre-welding heat treatment reduced the non-equilibrium segregation of boron during the cooling process, and it inhibited the formation of a liquid film at the grain boundary and liquefied cracking, thus improving the microhardness of the weld zone (WZ), and affecting the microhardness of the base metal (BM) and the heat-affected zone (HAZ). Panov et al. [23] studied the influence of a preheating and post-welding heat treatment on the microstructure and mechanical properties of the laser-welded joints of a Ti alloy. An increase in the preheating temperature led to an increase in the width of the WZ and HAZ, and an increase in the porosity and gaseous element content (i.e., O and N), while the microhardness of the joint was lower than that of the base material. Luo et al. [24] studied the application of a preheating treatment in an aluminum alloy 5052 welding plan. The experimental results showed that the contact resistance at the W/W (workpiece-to-workpiece) faying interface after the preheating was very consistent and could be reduced by two orders of magnitude. The uncertain variation in the contact resistance of the W/W mapping surface was almost reduced or eliminated, and the quality of the spot welding in terms of the peak load and nugget diameter was checked and showed great improvement.

In practical engineering, different types of thick plate members are usually welded together to produce complex structures and efficient industrial requirements; therefore, it is very important to predict and control the distribution of the thermal cycle curve and residual stress in the welding process to improve the design rationality and safety of actual engineering structures. In order to meet this requirement, this paper presents a new heating method before welding—i.e., ceramic sheet heating. The ceramic electric heater uses the heating element as the heat source when a workpiece is heated. The preheating method is mainly composed of a temperature control box, a caterpillar heater and a temperature measuring instrument. It can be widely used in the local heat treatment of various alloy steel-welded structures. The ceramic heating device changes the defects of the traditional process such as an unstable quality, high energy consumption and poor working conditions. The secondary development of the MIG welding process was carried out by the ABAQUS software. Three-dimensional modeling and pre-welding strengthening measures were carried out on welds under low temperature conditions, and important parameters such as an appropriate welding voltage, the welding current and welding speed were selected as the fixed values within a certain range. The changes in the thermal cycle, residual stress and deformation under two pre-welding heating methods were compared and analyzed, so as to understand the detailed welding process, and obtain the best welding simulation quality of the pre-welding heating method. The results of the simulation and welding test were compared and analyzed to provide a reliable theoretical basis for further research.

## 2. Experiment

**Figure 1:**
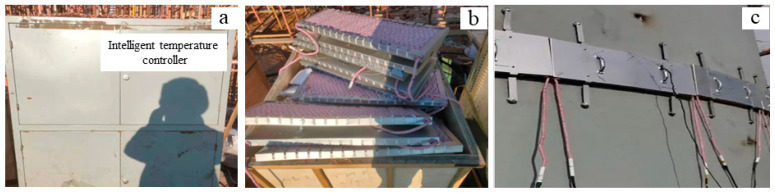
Ceramic heating-related device. (**a**) Temperature control box, (**b**) ceramic heating plate, and (**c**) ceramic heating construction.

Q345 structural steel is a Chinese, standard, low-alloy, medium-tensile strength steel produced by a hot rolling process. Due to its comprehensive mechanical properties and welding properties, it has been widely used in railways, bridges, industrial plants, boilers, pressure vessels, steel fuel tanks, power stations and other manufacturing structural components. The main microstructures of the Q345C steel used in the test were ferrite and pearlite, which has strong mechanical and fatigue properties such as strength, toughness and weldability [25]. Its chemical composition is shown in Table 1. After a high-temperature repeated cooling test [26], it was found that the Q345C steel had good mechanical properties when it was heated to 200–500 °C and cooled to the ambient temperature; however, with an increase in the circulating heating and cooling times and heating temperature, its microstructure changed significantly and its mechanical properties decreased significantly. The thick plate Q345C steel was welded under a traditional flame and ceramic plate heating, respectively, to study the influence of different preheating methods on welding stress and deformation. The ceramic heating device is shown in Figure 1. According to the heat treatment workpiece, the required crawler ceramic electric heater was connected with stainless steel wire (where the distance should not exceed the wall thickness), and then covered on the heating workpiece, then the ceramic electric heater was tied with stainless steel wire or a stainless steel belt. When welding the outside, the heater was arranged inside the furnace body. When welding the inside, the heater was arranged outside.

The ceramic heater has the following characteristics: (1) it has a high power density, can be rapidly heated, and its heating speed is much greater than for induction heating. (2) It has a small size, a simple and reasonable structure, and is light weight for light handling and dismantling labor. (3) The number of ceramic electric heaters can be determined according to the needs of a heat treatment work, and it is not bound by any conditions. (4) The ceramic heater is directly covered on a heat treatment workpiece, and the outer layer is covered with a layer of thermal insulation blanket (i.e., a needled blanket), which does not require any material with a large capacity, so that the heater’s heat loss is small, and the effect on energy saving is significant.

The thick plates under both preheating methods had the same geometric dimensions, i.e., 300 mm × 300 mm × 25 mm. Under the same welding parameters, the MIG welding process was performed on the two plates. In order to prevent lamellar tearing of the thick plate welding, gas-shielded welding was used for the welding.

**Figure 2:**
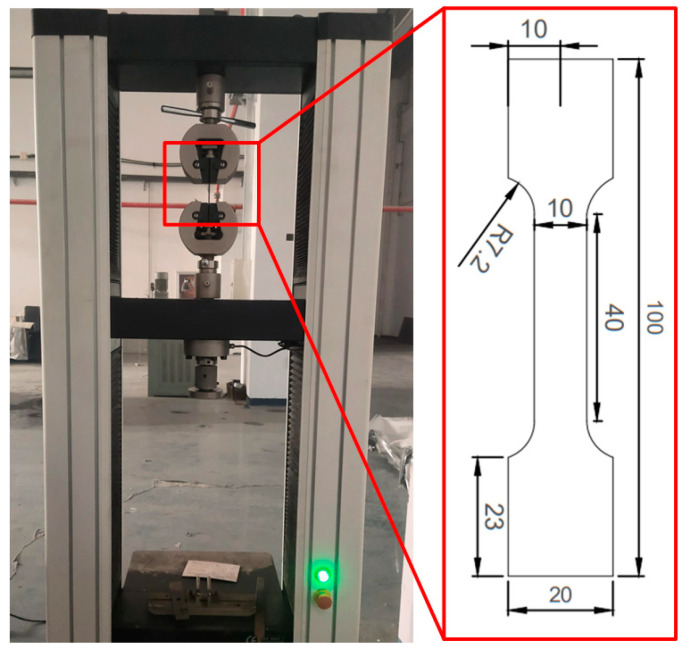
Tensile test through clamping specimens.

In order to make the finite element simulation correspond to the actual welding conditions of the butt-joint parts, the welded parts were prepared into tensile specimens to study the mechanical properties and microstructures of the welded joints. For the preparation of the tensile specimens, two tensile specimens obtained from the welded joints of thick plates under different heat treatment conditions were cut with a wire cutting machine. According to the relevant standards, the cross-sectional area of the sample was 10 mm × 2 mm. In addition, the sample was clamped to the SANS Electronic universal testing machine with a tensile rate of 1 mm/min, as shown in Figure 2.

At the same time, in order to analyze the changes in the mechanical properties and microstructures of T-welded joints under different welding currents or thermal inputs, and to study the influence of welding defects on the mechanical properties in detail, some measurements and sample preparations were carried out. For example, a sample for metallographic observation was cut with a wire-cut machine from a joint perpendicular to the welding direction and cold-set after wiping the sample. After grinding and polishing, the sample was etched in a nitric acid reagent for 5–10 s. Then, in order to clearly see the distribution of the welding defects and the chemical composition of the materials, the morphology and microstructure of the obtained metallographic samples were analyzed by optical microscopy (OM) and scanning electron microscopy (SEM).

## 3. Numerical Simulation Framework

### 3.1. Characteristics of Materials

**Figure 3:**
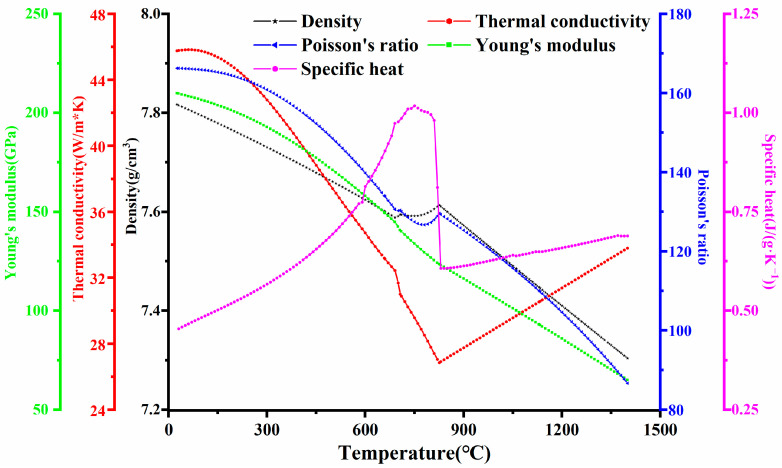
Temperature-dependent performance parameters of Q345C steels.

In the numerical simulation of the welding process, the temperature-dependent performance parameters of different welding zones are required, which are related to the chemical composition. According to the reference in [1] and the material property simulation software, JmatPro, the temperature-related performance parameters were given, as shown in Figure 3. For a welding temperature field analysis, the specific heat is important. In addition, it is necessary to determine the thermodynamic properties of the stress field simulation, such as via Poisson’s ratio and Young’s modulus.

In order to meet the requirements of the working conditions of the welded sheet at low temperature, the heating technology of a ceramic sheet before welding was proposed. The experimental sample was heated to 100 °C before welding and was then kept warm, then, the welding was compared with a traditional flame heated to 100 °C. In the analysis of the thermal cycle and stress field in the welding process, the model was simplified accordingly, and the assumption was that: (1) the fluid flow in the molten pool was Newtonian, incompressible, and a laminar flow; (2) all the molten pools and droplets were symmetric with the weld center axis; and (3) the arc axis in the welding process was straight; while (4) changes in the physical properties of the weld metal caused by mixing in the weld pool were not considered.

### 3.2. Finite Element Model

**Figure 4:**
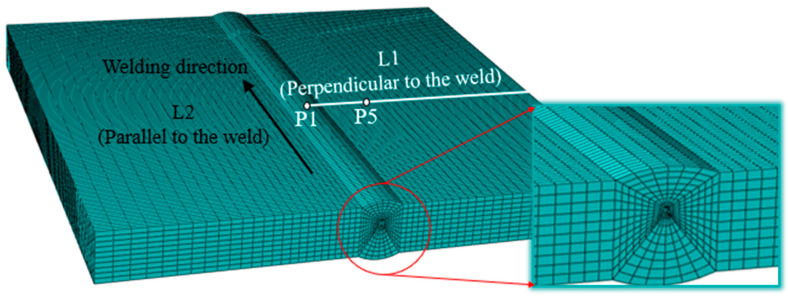
Finite element model meshing and simulated measurement position.

The butt joint was an X-type groove created with CO_2_ gas-shielded clean root welding, and then, the front and back with MIG filling welding. As the plate was of a medium thickness, it adopted three layers with three passes of welding. The welding process parameters of each weld are shown in Table 2. Firstly, the root was cleared. The upper groove opening angle was 60° for the double-layer double-pass welding, and the lower groove opening angle was 45° for the single-layer single-pass welding. A 3D welding model based on a finite element analysis was established. Figure 4 shows the meshing and boundary conditions of the welded finite element model, using C3D8R hexahedral elements and creating a fine mesh near the WZ for more accurate results. During the welding process, the welding parts and the heat source exchange heat with the surrounding medium through heat-transfer and heat-radiation. The heat radiation around the molten pool is dominant, and the convective heat-transfer away from the molten pool is dominant. The formula for calculating the heat flux loss is [11]:

(1) $$q_{s}=h_{c}\left(T_{s}-T_{0}\right)+\varepsilon \sigma [T_{s}^{4}-T_{0}^{4}]$$

where:

$q_{s}$—heat flux loss during welding;

$h_{c}$—coefficient of heat transfer;

$T_{s}$—the temperature of the welded part;

$T_{0}$—ambient temperature;

ε—surface emissivity, and for the high-strength steel Q345C, it was assumed to be 0.09;

σ—Stefan–Boltzmann constant.

Based on a thermal simulation, the stress field can be calculated according to the thermoelastoplastic theory. The calculated temperature field is used as the thermal load to calculate the stress field. Appropriate constraints must be applied during a thermal simulation. The boundary conditions of the welding fixture were completely constrained, and the lower surface of the weldment was constrained along the thickness direction. Because the project was carried out in a temperature retaining shed, the default initial ambient temperature was 5 °C.

### 3.3. Heat Source Model

**Figure 5:**
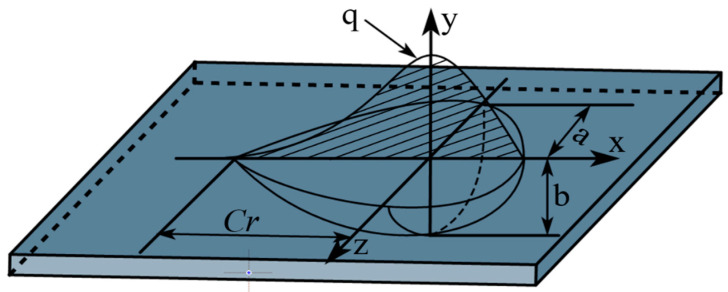
Double ellipsoid heat source distribution model.

By modeling the heat source, it was easy to determine the location of the heat field. A double ellipsoidal heat source distribution function was used as the heat source load, and the heat source distribution model is shown in Figure 5. The double ellipsoidal heat source distribution model (i.e., Goldak heat source) is a heat source model improved by Goldak on the basis of Gauss. The heat flow not only acts on the surface, but also has heat flow at a certain depth, namely, the volume heat source. Moreover, the heat flux is the Gaussian distribution in the width, length and depth directions. The influence of the arc stiffness was taken into account in this model, in which the welding method with a large arc impact is more accurate. In addition, if the depth of the molten pool is large, the change of the welding heat source in the depth direction needs to be considered, and the Goldak model is usually used in this case. It is assumed that the heat flux distribution on the body conforms to the double ellipsoid distribution, which can be expressed by the following formula [27]:

(2) $$q_{1}\left(x,y,z\right)=\frac{6\sqrt{3}(f_{1}Q)}{a_{1}bc\pi \sqrt{\pi }}e^{\left[-3(\frac{x^{2}}{{a_{1}}^{2}}+\frac{y^{2}}{b^{2}}+\frac{z^{2}}{c^{2}})\right]},x\geq 0$$

(3) $$q_{2}\left(x,y,z\right)=\frac{6\sqrt{3}(f_{2}Q)}{a_{2}bc\pi \sqrt{\pi }}e^{\left[-3(\frac{x^{2}}{{a_{1}}^{2}}+\frac{y^{2}}{b^{2}}+\frac{z^{2}}{c^{2}})\right]},x<0$$

(4) $$f_{1}+f_{2}=2.0$$

where:

$a_{1}$, $a_{2}$, b, c—the heat source shape parameter model;

Q—the effective power of the heat source;

$f_{1}$, $f_{2}$—the ratio of the heat source between the front and the back.

## 4. Results and Analysis

### 4.1. Thermal Cycle Curve Distribution

**Figure 6:**
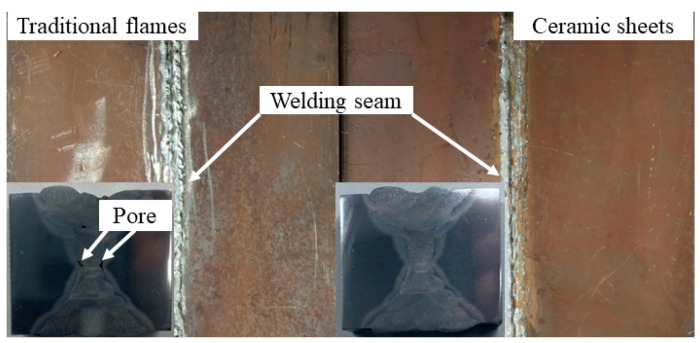
Welding results of Q345C steel under two heating methods.

**Figure 7:**
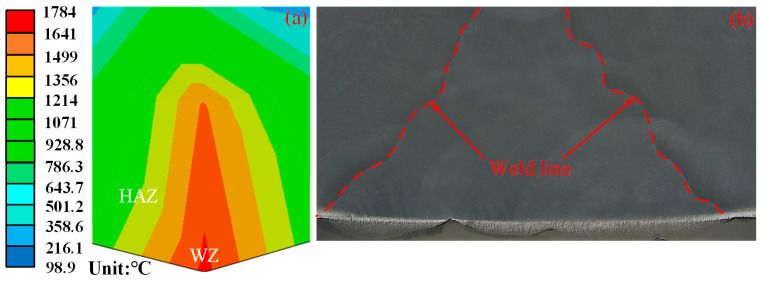
Temperature field of the welding seam of ceramic sheet under heating: (**a**) calculated weld geometry; (**b**) test weld geometry.

Figure 6 shows a thick plate Q345C steel welded under a conventional flame and ceramic sheet heating. With the MIG welding process, both kinds of plates had a good weld with a full penetration and a similar welding width due to the same welding parameters. The macro-geometric and micro-structural characteristics of different welding zones are related to the welding thermal cycle. According to the microstructure, there are three different zones in the weld including the WZ, the HAZ and the BM [28,29]. There was no WZ before the welding began because the WZ is formed by the continuous melting of welding wire. With the movement of the welding heat source, the welding began to produce a WZ and the whole WZ was formed until the end of welding. The welding process not only changed the geometric characteristics of the welded joints, but also changed the metallurgical and mechanical properties of materials in the different welding zones. Figure 7 shows the cross section outline of the weld. The cross section of the welded joint was divided into two typical zones, namely, the WZ and the HAZ. Through measurements, the width of the top of the WZ obtained by the simulation in Figure 7a and the experimental results in Figure 7b were 1.830 mm and 1.655 mm, respectively. The comparison of the results in Figure 6 shows that the experimental weld morphology was basically consistent with the simulated weld morphology.

**Figure 8:**
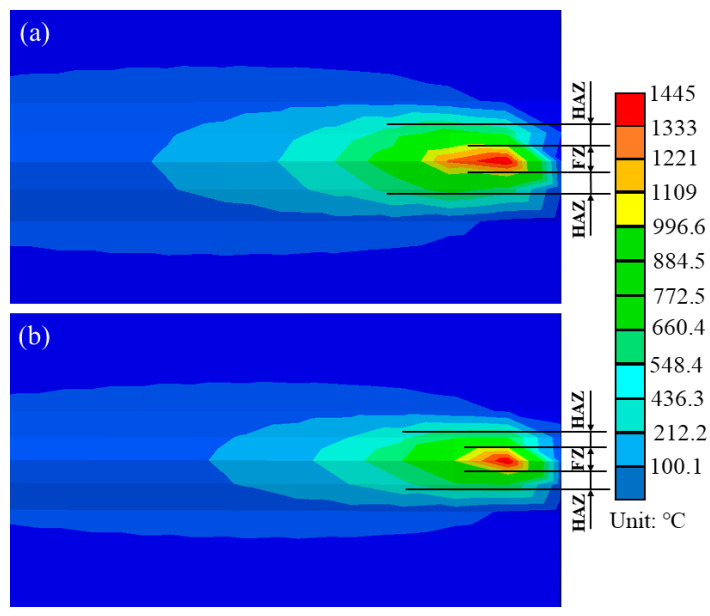
Numerical simulation results of the welding finite element temperature. (**a**) Conventional flame heating, and (**b**) ceramic sheet heating.

In order to study the influence of pre-welding heating of a ceramic sheet and flame on the welding process, the two simulated welding parameters were in the same operation, with the welding parameters shown in Table 2. Welding is a rapid heating and cooling process. In the heating stage, the metal material quickly melted, and after welding it quickly cooled. During this process, the material was first heated and expanded to create compressive stresses in the weld area. While during the cooling phase, the weld began to contract to create tensile stresses. The welding thermal cycle directly leads to the different mechanical properties of welds in different areas; therefore, the maximum temperature distribution helps to determine the geometric boundary of each welding area [30]. Figure 8 shows the 3D finite element numerical simulation results of the welding temperature under two working conditions under the same scale when the welding time was 468 s (i.e., the second weld). According to the peak temperature in the welding process in different areas, the cross section of the welded joint was divided into the three typical areas described above. Figure 8 shows the symmetrical geometric features of the WZ and HAZ on the upper surface of the workpiece along the welding direction. Figure 8a shows the simulated temperature field under traditional flame heating, and Figure 8b shows the simulated welding temperature field under ceramic plate heating. Where the liquid temperature of the WZ exceeded 1109 °C, the temperature of the HAZ was between the phase transition isotherm (1109 °C) and the solution temperature (660 °C). It could be seen that the size of the molten pool and the WZ obtained with the ceramic sheet heating welding was smaller and the area of the HAZ was reduced compared with the traditional flame heating welding method, which effectively improved the temperature gradient of the WZ.

**Figure 9:**
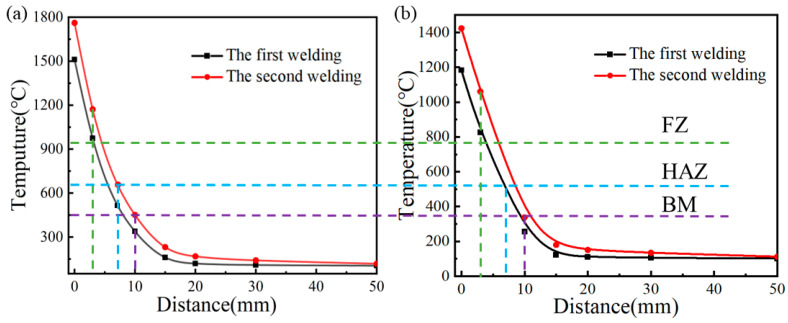
The highest temperature reached during welding. (**a**) Conventional flame heating, and (**b**) ceramic sheet heating.

In order to clarify the complex thermal cycle curve and stress-strain evolution mechanism in the welding process, the historical temperature data points of five points on and near the weld were extracted between P0–P4 on L1, as shown in Figure 4. The coordinates of the five points were P0 (150, 25, 150), P1 (160, 25, 150), P2 (170, 25, 150), P3 (180, 25, 150), and P4 (200, 25, 150). The point of P0 was located on the weld, and points P1–P4 were at some points adjacent to the weld at a certain distance. Figure 9 shows the maximum temperature reached by the nodes perpendicular to the weld during the welding process. It was found that the maximum temperature significantly decreased in the welding process and the curve dropped more gently, with a smaller temperature gradient. It can be noted that the ceramic sheet heating method will affect the maximum temperature of the molten pool and the temperature field behind the heat source when comparing it with the traditional flame heating method.

**Figure 10:**
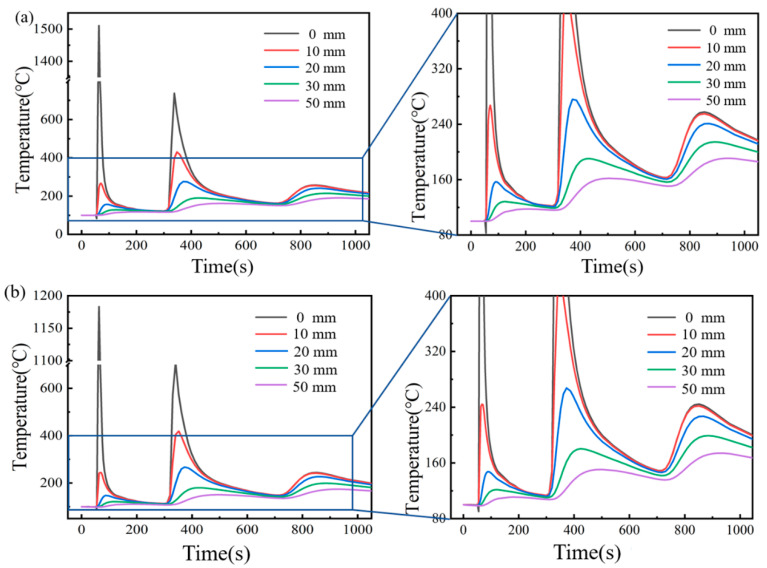
Heat cycle curve of characteristic points. (**a**) Conventional flame heating, and (**b**) ceramic sheet heating.

The temperature thermal cycle of three coordinate points from P0 to P4 is shown in Figure 10. For the five measuring points perpendicular to the welding direction, the maximum temperature decreased with an increasing distance from the WZ. As the welding heat source approached, the temperature at P0 increased rapidly. Although the molten pool expanded due to the increase in temperature, compressive plastic strain appeared in the welding area due to the restriction of the surrounding base metal. After the heat source had passed through P0, the temperature at the point dropped rapidly, and the welding area solidified and shrunk. Due to the metal limitation around the molten pool, the tensile longitudinal plastic strain was generated after cooling, which cancelled out the compressive plastic strain during the heating process [31]; therefore, after the laser heat source had passed through P0, the longitudinal plastic strain decreased rapidly and reached a constant value. For P0, when the heat source passed by the point through the two heating methods of a traditional flame and ceramic sheet, there was an obvious difference between the maximum temperature of P0 in the process of the three-welds welding. When the first weld was welded, the maximum temperature under traditional flame heating was 1500 °C, and the maximum temperature of the ceramic sheet heating was 1180 °C, which decreased by 21.33%. In the process of the second welding, the maximum temperature of the heat source passing through the point under the two heating methods was 750 °C and 580 °C, respectively, which decreased by 22.67%. The temperature variation trend of the four points of P1 to P4 in the process of the three-pass welding was similar to P0.

### 4.2. Residual Stress Distribution

**Figure 11:**
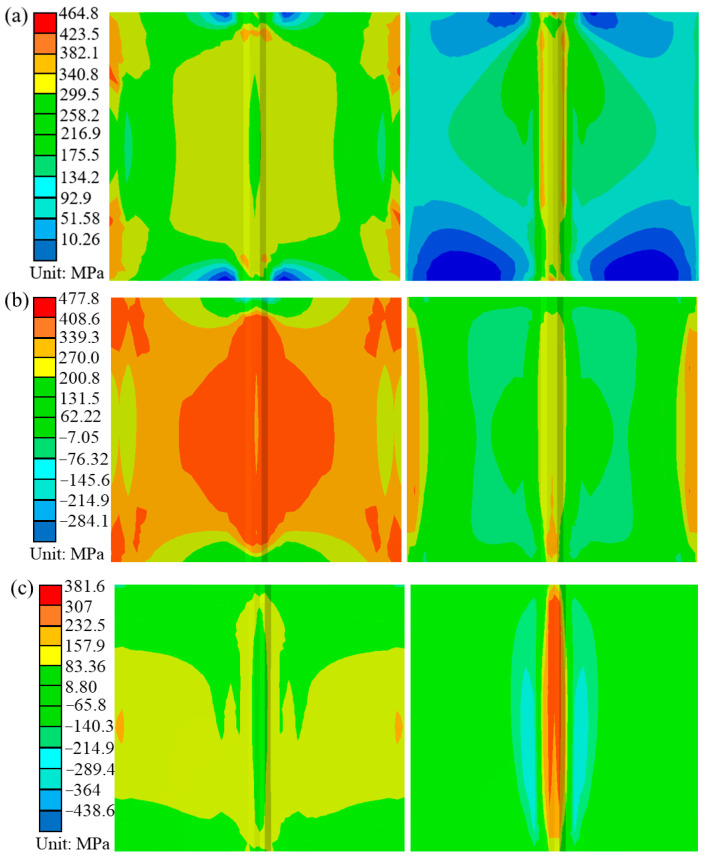
Residual stress distribution after welding. (**a**) Overall residual stress, (**b**) longitudinal residual stress, and (**c**) transverse residual stress.

Figure 11 shows the contours of the global, longitudinal and transverse residual stresses under the pre-welding heating of a conventional flame and ceramic sheet, respectively. By comparison, it can be found that the selection of the pre-welding heating method has great influence on the distribution of the overall residual stress, transverse and longitudinal residual stress. Although the distribution of residual stress was similar in the two cases, the magnitude of the residual stress was very different. It can be seen from Figure 11a that the equivalent residual stress near the weld of the weldment and the whole sheet under the traditional flame heating was larger, and the maximum equivalent residual stress was 464.8 MPa. The ceramic heating method changed this phenomenon and reduced the area of the equivalent residual stress region. The maximum residual stress of the welded specimens decreased significantly to 404.7 MPa, down by 12.93%. If the end effect of the weld was ignored, the equivalent residual stress in the middle of the weld was evenly distributed. Figure 11b shows the longitudinal residual stress distribution of the welded parts heated by s traditional flame and ceramic sheet, which indicated that the longitudinal stress of the weld and its vicinity was tensile stress. Because the filler wire and the base metal underwent rapid heating and cooling during the welding process, the tensile residual stress reached the yield strength of the base metal [32]. As the distance from the weld increased, the tensile stress gradually became compressive stress [33]. Figure 11c compares the transverse residual stress distribution of the two samples. The contour lines in the figure show that the transverse residual stress had a similar distribution in both cases. The transverse residual stress was high-tensile stress near the weld and WZ. With the increase in the distance from the weld, the tensile stress decreased gradually, and the transverse tensile stress weakened gradually. At the same time, the transverse residual stress near the weld under the heating of the ceramic sheet became the compression stress with a low value. It can be clearly compared from the above three figures that the residual stress was obviously improved by heating the ceramic sheet. Compared with the transverse residual stress, the longitudinal residual stress was more significantly affected by the two pre-welding heating methods.

**Figure 12:**
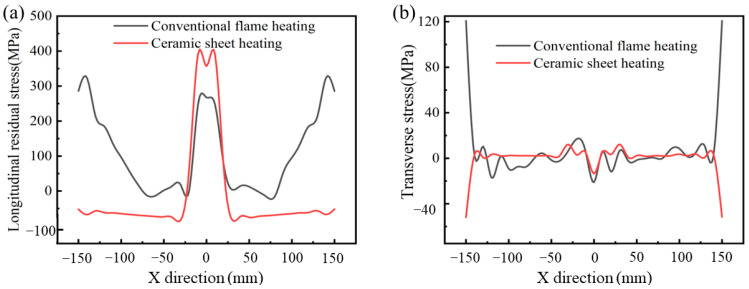
Residual stress distribution along the direction perpendicular to the weld. (**a**) Longitudinal residual stress, and (**b**) transverse residual stress.

Figure 12 shows the longitudinal and transverse residual stresses of the butt weld calculated by the finite element model along the direction perpendicular to the weld under the two pre-welding heating conditions. It is obvious that the residual stress perpendicular to the welding direction was symmetrically distributed along the weld center. For Figure 12a, the longitudinal residual stress near the WZ and HAZ was tensile stress. The peak stress under the conventional flame heating was about 270 MPa, while the peak stress under the ceramic plate heating was about 400 MPa. Although the heating stress of the ceramic sheet near the weld and HAZ was higher than that of the traditional flame, the longitudinal residual stress was significantly reduced and evenly distributed in the area outside until it gradually approached zero at the free surface of the end edge [34]. Figure 12b shows the transverse residual stress perpendicular to the weld, which was almost uniformly distributed along the *X*-axis, and the overall transverse residual stress was basically zero. It can be clearly seen that the transverse residual stress after welding was obviously reduced and distributed evenly with minimal fluctuation. In addition, the longitudinal residual stress was much greater than the transverse residual stress, namely, about ten times greater than the transverse residual stress, which may be the main cause of the plate buckling. The longitudinal stress was basically tensile stress, which was due to strict constraints. Due to strict constraints, the longitudinal stress had a high-tensile value near the weld and became compressed away from the weld, and the transverse residual stress distribution was almost constant relative to the longitudinal stress distribution. This made the longitudinal stress basically a tensile stress [35]. It can be concluded that most of the stress in the sample was mainly caused by the longitudinal stress and was mainly concentrated in the WZ.

**Figure 13:**
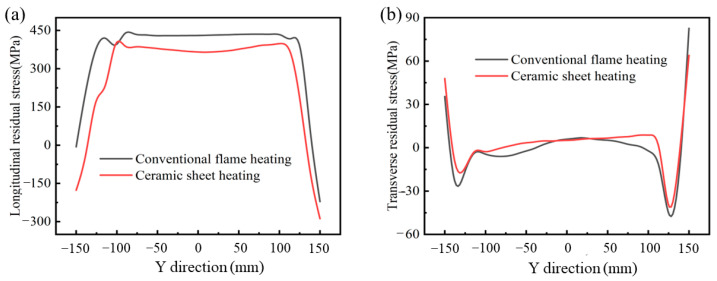
Residual stress distribution along the direction parallel to the weld. (**a**) Longitudinal residual stress, and (**b**) transverse residual stress.

The residual stress along the welding direction is shown in Figure 13. The longitudinal stress of the two pre-welding methods was negative at the beginning and end of the weld, indicating the existence of compressive residual stress, which gradually increased along the length of the weld and became tensile. Within the range of 50~250 mm in the direction of the weld, there was a stable high-tensile stress zone, in which the maximum longitudinal residual stress reached 450 MPa, as shown in Figure 13a. As a result, the welded joint was subjected to significant tensile stress and deformation along the length of the weld. As shown in Figure 13b, the transverse residual stress along the weld was basically zero and the stress value increased gradually away from the weld, indicating that a large tensile stress had been generated due to the constraint effect of the edge. Compared with the residual stress perpendicular to the weld direction, the residual stress along the weld direction was less affected by the pre-welding heating method, but it still had a slightly improved effect. It can be seen from the above analysis that, compared with the traditional flame, the heating method of the ceramic sheet had a significant weakening effect on the residual stress of the weldment. Its tensile residual stress was significantly reduced, and especially, the effect on the residual stress perpendicular to the weld direction was more significant. The lower tensile residual stress reduced the risk of failure under a cyclic or dynamic load [36].

### 4.3. Deformation

**Figure 14:**
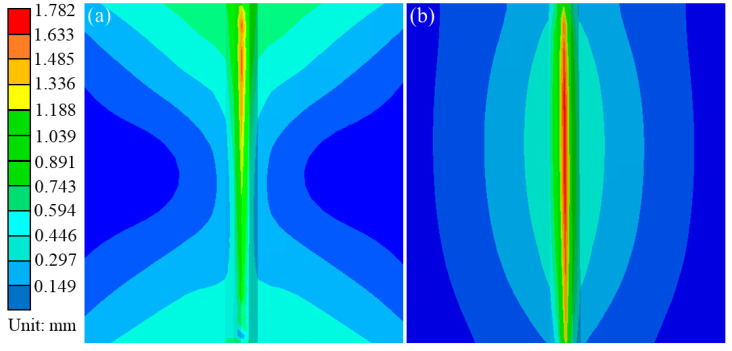
Distribution of deformation after welding. (**a**) Conventional flame heating, and (**b**) ceramic sheet heating.

**Figure 15:**
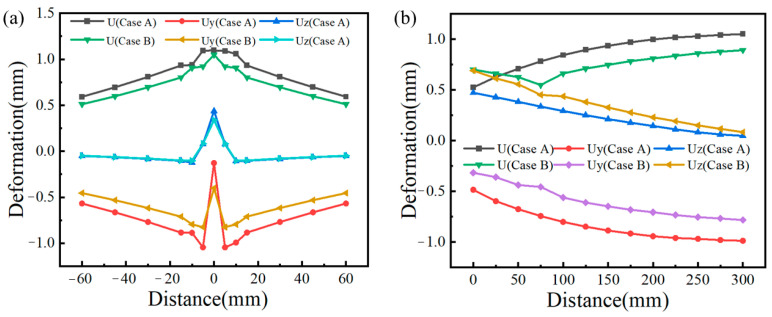
Welding deformation curves under different heating methods. (**a**) Perpendicular to the weld direction (L1), and (**b**) parallel to the weld direction (L2).

Similarly, in order to analyze the influence of traditional flame and ceramic sheet heating on welding deformation, the feature points as shown in Figure 4 were extracted in directions perpendicular to the weld and parallel to the weld, respectively. Figure 14 describes the out-of-plane deformation curves of L1 and L2 lines under different cold source distances (Case A is the traditional flame heating mode, and Case B is the ceramic sheet heating mode). As can be seen from Figure 14, under these two conditions, the maximum total post-welding deformation of the ceramic sheet heating was 1.782 mm, which increased by 9.06% compared with the maximum welding deformation of 1.634 mm under the traditional flame heating. Although the deformation on the weld increased in a concentrated way, the total deformation at both ends of the weld surface had an obvious decreasing trend and the regional distribution of the overall deformation also decreased obviously, while the deformation after welding was improved. Generally speaking, the smaller the binding force during welding, the smaller the welding displacement. Although the binding force is the main cause of welding deformation, the bending deformation caused by a temperature gradient in the thickness and width direction of a sample is a necessary factor to cause buckling and to influence welding deformation [37]. In order to clarify the cause of this phenomenon in detail, the displacement data perpendicular to and along the welding direction (L1) in Figure 4 were extracted. As shown in Figure 15a, the total deformation, and the *Y*-axis and *Z*-axis deformation curves perpendicular to the weld direction were symmetrically distributed with the weld as the axis of symmetry. The deformation near the weld was the largest, and the amount of deformation away from the weld decreased gradually, which may be attributed to the temperature gradient. Relative to the total deformation and the *Z*-axis deformation, the *Y*-axis deformation was almost unchanged. Similarly, the welding deformation along the weld (L2) direction is shown in Figure 15b, where the welding deformation varied in a certain proportion. The total deformation parallel to the weld (L2) was significantly reduced when the ceramic sheet was heated compared with the traditional flame welding, indicating that the pre-welding heating method of the ceramic sheet can effectively reduce or mitigate welding buckling in the welded joint.

### 4.4. Welding Results and Microstructure

According to the tensile results of the base metal and butt-welded joint in the experimental description above, the yield strength and tensile strength are shown in Table 3. It can be clearly seen from the ratio of the strength of the welded joint to the strength of BM that the yield strength and tensile strength were significantly lower than that of the base material. In order to better study the cause of this result and to analyze the difference of the microstructure of the weld under two heat treatment methods, the metallography and microhardness of the butt-welded joint were measured, and the changes in the microstructure and microhardness of the joint were analyzed.

**Figure 16:**
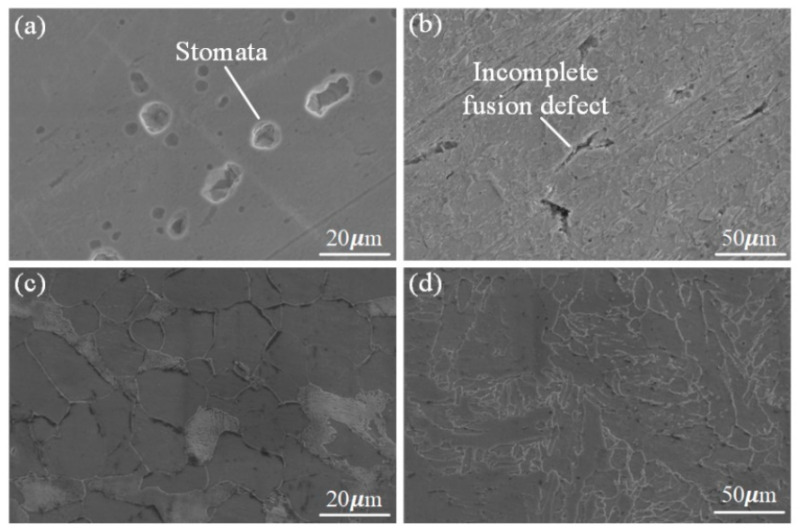
Weld of the WZ and HAZ under SEM; (**a**,**b**) flame heating mode; (**c**,**d**) ceramic sheet heating method.

**Figure 17:**
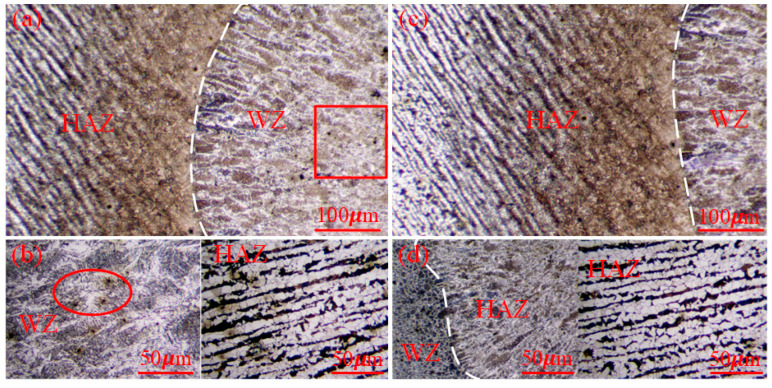
Microstructure features of the WZ and HAZ; (**a**,**b**) flame heating mode; (**c**,**d**) ceramic sheet heating method.

The weld microstructure and grain boundary morphology of the WZ and HAZ under the different heat treatments are shown in Figure 16. A metallographic observation was carried out using OM to obtain the microstructure characteristics of the BM to the WZ, as shown in Figure 17. Combined with the two figures, it is clear that there were some welding defects in the butt joint under the flame heat treatment mode, such as an incomplete fusion defect and a porosity defect of the WZ and HAZ. Welding defects have a great influence on the tensile strength, especially porosity defects, which are important factors affecting the tensile properties.

In Figure 17, due to the obvious porosity defect in the WZ under flame heating, one of the reasons for this is that the yield strength and tensile strength of the butt joint were somewhat lower than that of the base material; however, the weld strength after the heat treatment of the ceramic sheet was higher than that obtained by the flame heating, and no obvious porosity and cracks were observed in the FZ or the weld edge. Figure 17a,c represents the evolution of the microstructure of the welded joint from the HAZ to the weld center. It was composed of the BM, HAZ (i.e., a mixed zone of equiaxed dendrites and equiaxed grains with a coarse rolling-aged microstructure) and WZ. There were some differences in the microstructure characteristics of the WZ and HAZ. Due to the large temperature gradient near the weld, a clear columnar grain structure could be observed at the boundary of the WZ, which made the grain grow along the solidification front [38], resulting in different grain sizes. In the HAZ near the center of the weld, although the temperature gradient in the weld decreased, the higher temperature provided conditions for the growth of grain, which made the ferritic and pearlite of the base metal grow, while the distance narrowed.

## 5. Conclusions

In this paper, the influence of two kinds of pre-welding heating methods of, namely, a traditional flame and a ceramic sheet, on the thermal cycle, residual stress and deformation after welding of a Q345C-steel, thick plate was studied by the finite element theory, which can be used to improve the mechanical properties of the butt joint weld and improve the structural strength and service life of a whole component. The effects of the different heating methods on the welding evolution were studied by simulation and an experimental verification was carried out. The main conclusions of this study are as follows:

(1) A new heating method before welding was proposed. The method used a ceramic electric heater composed of heating elements as the heating source before welding. Compared with the traditional flame heating method, this welding method is controllable, relatively safe and of low cost. The weld shape was basically the same as the simulated weld shape and the quality was good.
(2) A three-layer and three-pass welding model of the thick plate weldment was established. The results show that the experimental weld morphology was basically consistent with the simulated weld morphology. It showed that although the pre-welding heating method had little influence on the welding temperature field, the maximum temperature of the node affected by the welding process was significantly reduced, and the maximum temperature difference between the two at the same characteristic point was 170 °C. The heating mode of the ceramic sheet affected the maximum temperature of the molten pool and the temperature field behind the heat source, and it effectively improved the thermal cycle curve.
(3) The influence of the two heating methods on the reduction or even elimination of the residual stress and deformation caused by welding was analyzed. The results show that the ceramic heating had a significant weakening effect on the residual stress of the welded parts, and especially, the effect on the residual stress perpendicular to the weld direction was more significant, which decreased by 5.88%.
(4) The welding deformation under the heating mode of the ceramic plate was significantly less than that under the traditional flame heating, and especially, the weakening effect of the deformation parallel to the weld direction was more obvious. The maximum total post-welding deformation of the ceramic sheet after heating was 1.782 mm, which was 9.06% higher than the maximum welding deformation of 1.634 mm under the traditional flame heating.
(5) The microcosmic experiment showed that the ceramic heating method produced a better strength and joint quality. In the flame heat treatment, the butt joint had some welding defects, such as incomplete fusion defects, and WZ and HAZ porosity defects. The weld strength of the ceramic sheet after the heat treatment was higher than that after the flame heating, while there were no obvious pores and cracks in the weld zone and weld edge, and it had a better tensile strength.

## Figures and Tables

**Table 1:** The chemical composition of Q345C steel.

Composition (%)	C	Mn	Si	S	P	Cr	Mo	V	Cu	Ni
	0.15	1.44	0.23	0.005	0.013	0.025	0.0077	0.031	0.016	0.0095

**Table 2:** Welding process parameters.

Parameters	Unit	Welding		
		1	2	3
Voltage of welding	V	21	22	23
Current of welding	A	110	115	120
Power of welding	W	1733	1898	2070
Speed of welding	mm/s	0.1		

**Table 3:** Mechanical properties of the welded joint.

Heat Treatment Method	Yield Strength (MPa)	Tensile Strength (MPa)
Flame heating	503	523
Ceramic heating	542	569
BM	587	612

## Data Availability

No data or materials are available for this research.

## References

[1] Yang N., Su C., Wang X.-F., Bai F. (2016). Research on damage evolution in thick steel plates. J. Constr. Steel Res..

[2] Zhang X., Wu L., Andrä H., Gan W., Hofmann M., Wang D., Ni D., Xiao B., Ma Z. (2019). Effects of welding speed on the multiscale residual stresses in friction stir welded metal matrix composites. J. Mater. Sci. Technol..

[3] Lee J.-H., Jang B.-S., Kim H.-J., Shim S.H., Im S.W. (2020). The effect of weld residual stress on fracture toughness at the intersection of two welding lines of offshore tubular structure. Mar. Struct..

[4] James M.N. (2011). Residual stress influences on structural reliability. Eng. Fail. Anal..

[5] Adamkowski A., Lewandowski M. (2015). Analytical model of stress concentration for the welded joints with angular distortion of thin-walled pipelines. Thin-Walled Struct..

[6] Sadeghian B., Taherizadeh A., Atapour M. (2018). Simulation of weld morphology during friction stir welding of aluminum- stainless steel joint. J. Mater. Process. Technol..

[7] Barsoum Z., Lundbäck A. (2009). Simplified FE welding simulation of fillet welds–3D effects on the formation residual stresses. Eng. Fail. Anal..

[8] Zeng P., Gao Y., Lei L.P. (2009). Welding process simulation under varying temperatures and constraints. Mater. Sci. Eng. A.

[9] Yegaie Y.S., Kermanpur A., Shamanian M. (2010). Numerical simulation and experimental investigation of temperature and residual stresses in GTAW with a heat sink process of Monel 400 plates. J. Mater. Process. Technol..

[10] Islam M., Buijk A., Rais-Rohani M., Motoyama K. (2014). Simulation-based numerical optimization of arc welding process for reduced distortion in welded structures. Finite Elem. Anal. Des..

[11] Ai Y., Jiang P., Shao X., Li P., Wang C., Mi G., Geng S., Liu Y., Liu W. (2017). The prediction of the whole weld in fiber laser keyhole welding based on numerical simulation. Appl. Therm. Eng..

[12] Kik T., Moravec J., Novakova I. (2019). Numerical simulations of X22CrMoV12-1 steel multilayer welding. Arch. Metall. Mater..

[13] Chen G., Shu X., Liu J., Zhang B., Zhang B., Feng J. (2018). Investigation on microstructure of electron beam welded WC-Co/40Cr joints. Vacuum.

[14] Bal K.S., Dutta Majumdar J., Roy Choudhury A. (2019). Effect of electron beam accelerating voltage on the melt zone area, secondary-dendrite arm spacing and fusion line microstructure of bead-on-plate welded Hastelloy C-276 sheet. Optik.

[15] Cho D.-W., Cho W.-I., Na S.-J. (2014). Modeling and simulation of arc: Laser and hybrid welding process. J. Manuf. Process..

[16] He G.-Q., Yang Z.-G., Xi B., Zhang Y.-P., Wang D., Liu B., Li S. (2021). Deformation control during the welding of AP1000 main pump casing and steam generator. Nucl. Mater. Energy.

[17] Zhao M.S., Chiew S.P., Lee C.K. (2016). Post weld heat treatment for high strength steel welded connections. J. Constr. Steel Res..

[18] Li S., Xu W., Xiao G., Zhou Z., Su F., Feng J. (2020). Effects of Sc on laser hot-wire welding performance of 7075 aluminum alloy. Mater. Res. Express.

[19] Heinze C., Schwenk C., Rethmeier M. (2012). The effect of tack welding on numerically calculated welding-induced distortion. J. Mater. Process. Technol..

[20] Palanisamy V., Solberg J.K., Salberg B., Moe P.T. (2021). Weld thermal simulation of API 5CT L80 grade steel. Weld. World.

[21] Köse C. (2022). Heat treatment and heat input effects on the dissimilar laser beam welded AISI 904L super austenitic stainless steel to AISI 317L austenitic stainless steel: Surface, texture, microstructure and mechanical properties. Vacuum.

[22] Ola O.T., Ojo O.A., Chaturvedi M.C. (2014). On the development of a new pre-weld thermal treatment procedure for preventing heat-affected zone (HAZ) liquation cracking in nickel-base IN 738 superalloy. Philos. Mag..

[23] Panov D., Naumov S., Stepanov N., Sokolovsky V., Volokitina E., Kashaev N., Salishchev G., Ventzke V., Dinse R., Riekehr S. (2022). Effect of pre-heating and post-weld heat treatment on structure and mechanical properties of laser beam-welded Ti2AlNb-based joints. Intermetallics.

[24] Luo Z., Ao S., Chao Y.J., Cui X., Li Y., Lin Y. (2015). Application of Pre-heating to Improve the Consistency and Quality in AA5052 Resistance Spot Welding. J. Mater. Eng. Perform..

[25] Guo L., Zhang X.-Z., Feng C.-X. (2017). Continuous bending and straightening technology of Q345c slab based on high-temperature creep deformation. J. Iron Steel Res. Int..

[26] Zhang C., Gong M., Zhu L. (2022). Post-fire mechanical behavior of Q345 structural steel after repeated cooling from elevated temperatures with fire-extinguishing foam. J. Constr. Steel Res..

[27] Dai W.-H., Song Y.-T., Xin J.-J., Fang C., Wei J., Wu J.-F. (2020). Numerical simulation of the ITER BTCC prototype case enclosure welding. Fusion Eng. Des..

[28] Lezaack M.B., Simar A. (2021). Avoiding abnormal grain growth in thick 7XXX aluminium alloy friction stir welds during T6 post heat treatments. Mater. Sci. Eng. A.

[29] Kumar U., Gope D.K., Srivastava J.P., Chattopadhyaya S., Das A.K., Krolczyk G. (2018). Experimental and Numerical Assessment of Temperature Field and Analysis of Microstructure and Mechanical Properties of Low Power Laser Annealed Welded Joints [J/OL]. Materials.

[30] Wang J., Chen X., Yang L., Zhang G. (2022). Sequentially combined thermo-mechanical and mechanical simulation of double-pulse MIG welding of 6061-T6 aluminum alloy sheets. J. Manuf. Process..

[31] Chen L., Zhang Y., Xue X., Wang B., Yang J., Zhang Z., Tyrer N., Barber G.C. (2022). Investigation on shearing strength of resistance spot-welded joints of dissimilar steel plates with varying welding current and time. J. Mater. Res. Technol..

[32] Wu T., Ma Y., Xia H., Geng P., Niendorf T., Ma N. (2022). Measurement and simulation of residual stresses in laser welded CFRP/steel lap joints. Compos. Struct..

[33] Yang B., Lin D., Xia H., Li H., Wang P., Jiao J., Chen X., Tan C., Li L., Wang Q. (2022). Welding characterization evolutions for dual spot laser welded-brazed Al/steel joint with various spot configurations. J. Mater. Res. Technol..

[34] Muaz M., Choudhury S.K. (2019). Experimental investigations and multi-objective optimization of MQL-assisted milling process for finishing of AISI 4340 steel. Measurement.

[35] Keränen L., Nousiainen O., Javaheri V., Kaijalainen A., Pokka A.-P., Keskitalo M., Niskanen J., Kurvinen E. (2022). Mechanical properties of welded ultrahigh-strength S960 steel at low and elevated temperatures. J. Constr. Steel Res..

[36] Lee C.-H., Chang K.-H., Van Do V.N. (2015). Finite element modeling of residual stress relaxation in steel butt welds under cyclic loading. Eng. Struct..

[37] Chen G., Xue W., Jia Y., Shen S., Liu G. (2020). Microstructure and mechanical property of WC-10Co/RM80 steel dissimilar resistance spot welding joint. Mater. Sci. Eng. A.

[38] Wei H.L., Elmer J.W., Debroy T. (2017). Crystal growth during keyhole mode laser welding. Acta Mater..

